# Interpretation of health-related quality of life outcomes in Parkinson’s disease from the EARLYSTIM Study

**DOI:** 10.1371/journal.pone.0237498

**Published:** 2020-08-21

**Authors:** Pablo Martinez-Martin, Guenther Deuschl, Lisa Tonder, Alfons Schnitzler, Jean-Luc Houeto, Lars Timmermann, Joern Rau, Carmen Schade-Brittinger, Valerie Stoker, Marie Vidailhet, Paul Krack, on behalf of the EARLYSTIM Study Group

**Affiliations:** 1 Center for Networked Biomedical Research in Neurodegenerative Diseases (CIBERNED), Carlos III Institute of Health, Madrid, Spain; 2 Department of Neurology, UKSH, Kiel Campus Christian-Albrechts-University, Kiel, Germany; 3 Medtronic, Minneapolis, Minnesota, United States of America; 4 Department of Neurology, Institute of Clinical Neuroscience and Medical Psychology, Heinrich-Heine University Duesseldorf, Duesseldorf, Germany; 5 Department of Neurology, CIC-INSERM 1402, CHU of Poitiers, University of Poitiers, Poitiers, France; 6 Universitätsklinikum Giessen und Marburg, Marburg Campus, Marburg, Germany; 7 The Coordinating Center for Clinical Trials, Philipps University, Marburg, Germany; 8 Department of Neurology, Sorbonne Université, ICM UMR1127, INSERM &1127, CNRS 7225, Salpêtriere University Hospital, AP-HP, Paris, France; 9 Department of Neurology, University Hospital Bern, University of Bern, Bern, Switzerland; CNRS, FRANCE

## Abstract

The EARLYSTIM Study compared deep brain stimulation (DBS) with best medical treatment (BMT) over 2-years, showing a between-group difference of 8.0 from baseline in favor of DBS in health-related quality of life (HRQoL), measured with the PDQ-39 SI (summary index). This study obtained complementary information about the importance of the change in HRQoL as measured by the PDQ-39, using anchor-based (Patient Global Impression of Change, PGIC) and distribution-based techniques (magnitude of change, effect size, thresholds, distribution of benefit) applied to the EARLYSTIM study data. Anchor-based techniques showed a difference follow-up–baseline for patients who reported “minimal improvement” of -5.8 [-9.9, -1.6] (mean [95%CI]) in the DBS group vs -2.9 [-9.0, 3.1] in the BMT group. As the vast majority (80.8%) of DBS patients reported “much or very much improvement”, this difference was explored for the latter group and amounted to -8.7 for the DBS group and -6.5 in the BMT group. Distribution-based techniques that analyzed the relative change and treatment effect size showed a moderate benefit of the DBS on the HRQoL, whereas a slight worsening was observed in the BMT group. The change in the DBS group (-7.8) was higher than the MIC (Minimally Important Change) estimated value (-5.8 by the anchor; -6.3 by triangulation of thresholds), but not in the BMT (0.2 vs. -3.0 to -5.4, respectively). Almost 90% of the patients in the DBS group declared some improvement (58.3% and 56.7% beyond the estimated MIC), which was significantly different from the BMT group whose proportions were 32.0% and 30.3%, respectively. The number needed to treat to improve ≥1 MIC by DBS vs BMT was 3.8. Change in depression, disability and pain influenced the improvement of the DBS group. DBS improved HRQoL in a high proportion of patients to a significant and moderate degree, at 2 years follow-up.

## Introduction

Parkinson’s disease (PD) is a neurodegenerative disorder, second in prevalence after Alzheimer disease in population greater than 60 years, and the global burden of Parkinson’s disease has more than doubled with aging of the population and longer disease duration [1]. The semiology of PD includes characteristic motor manifestations (bradykinesia, rest tremor, rigidity, gait disturbances, and impairment of postural reflexes) and a variety of non-motor symptoms (e.g., sleep, mood, and autonomic disorders). Progression of the disease over time bears progressive disability, physical and mental complications (e.g., dyskinesia, dementia), psychosocial malfunction, and potential personal financial loss. All these factors can impact on and severely deteriorate the patients’ health-related quality of life (HRQoL) [2–6].

HRQoL is a component of the global QoL and may be defined as “the perception and evaluation, by patients themselves, of the impact that the disease and its consequences have caused in their life” [7]. The main components of the construct HRQoL are: Physical symptoms, Mental symptoms (mood and cognition), Functional ability, and Social functioning [8, 9]. Derived from traditions such as the health and social indicators, and designed and validated through psychometric theories, HRQoL measures are available. These measures can be classified as “generic”, usable in any condition or population, and “specific” for populations with specific characteristics, symptoms, condition, or dysfunctions. Generally biometrical presentation of results does not reflect the changes or statistical distribution-based views leading to limitations in understanding which changes matter to the patient. The information provided by the analysis and interpretation of the HRQoL measures provides relevant information about the impact of the disease, its course over time, priority areas to be attended, and effect of the interventions. Therefore, the perspective of the disease from the patients’ point of view is invaluable complementary information for a clinical practice and research, as recognized by the regulatory agencies [10, 11]. Measures used for HRQoL assessment in PD have been reviewed by an ad hoc Movement Disorder Society Task Force [12].

The EARLYSTIM Study was a prospective randomized study comparing subthalamic nucleus deep brain stimulation (DBS) with best medical treatment (BMT) to BMT alone over a 2-year follow-up. The primary endpoint was HRQoL as measured with the Parkinson’s Disease Questionnaire-39 items (PDQ-39) [13–20]. The “positive” results of the EARLYSTIM Study favor the use of DBS in PD patients with early motor complications. Consequently, the Food and Drug Administration (FDA) approved in November 2015 the use of DBS in PD patients with “at least 4 years duration and with recent onset motor complications, or motor complications of longer- standing duration that are not adequately controlled with medication”. The proposal of earlier intervention and the FDA approval, however, have not been free of criticisms [21–23].

In the pivotal paper of the EARLYSTIM study [14] the results of the primary endpoint focused on the difference (comparison) pre- post-intervention and the percentage of change.

We now address the clinical importance of the change, the relationships between change in the HRQoL and change in clinical aspects, and the proportion of patients experiencing a significant improvement by the intervention. Therefore, the objectives of this secondary analysis were: (1) to determine a set of parameters that allow translation to a pragmatic and clear meaning (i.e. negligible, mild, moderate, important, or very important) for the HRQoL outcomes of the EARLYSTIM Study, and (2) to assess what modifications in the manifestations of the disease were associated with changes in HRQoL.

## Methods

### Design

Post hoc secondary analysis of the EARLYSTIM data (baseline and 24 months).

### Population

Cohort of PD patients included in the EARLYSTIM Study which has been succinctly described as PD patients under age 61 with mild levodopa-responsive PD (motor response ≥50%), Hoehn & Yahr stage ≤2.5, and preserved psychosocial competence who experienced levodopa-induced motor complications for no more than 3 years [13].

### Assessments

The outcome assessments evaluated in this analysis were the following: Unified Parkinson’s Disease Rating Scale (UPDRS) sections II, III, and IV; a gait subscore derived from the UPDRS (sum of the scores from items 27, 28, 29, and 30), motor diary, VAS for Pain, Starkstein’s Apathy Scale, Scales for Outcomes in Parkinson’s Disease-Psychosocial (SCOPA-PS) for psychosocial adjustment, Social- and Occupational Functioning Assessment Scale (SOFAS), Beck Depression Inventory. Clinical Global Impression of Change (CGIC) and Patient Global Impression of Change (PGIC) were also assessed (the latter considered more appropriate to compare with a patient-reported outcome), and HRQoL was evaluated with MOS Short Form 36 items (SF-36), and PDQ-39 Summary Index (PDQ-39 SI). Note, that an increase in the PDQ-39 SI value means HRQoL worsening whereas a decrease means improvement.

### Ethics statement

The EARLYSTIM study conformed to the Declaration of Helsinki and all patients provided written informed consent before randomization. Permission was approved by the local ethics committees of all centers (Ethik-Kommission, Medizinische Fakultät der Christian-Albrechts-Universität zu Kiel, Kiel, Germany; Comité de Protection des Personnes, Ile de France IV, Hôpital Saint-Louis, Paris, France). The study was registered at ClinicalTrials.gov (NCT00354133).

### Analysis methods

This analysis used the data from the EARLYSTIM Study and focused on HRQoL outcomes interpretability. The variable of interest was the change observed in the PDQ-39 SI at a 24-month follow-up (FU).

### Objective 1: Quantifying the meaning of HRQoL outcomes

#### 1. Anchor-based method

The Minimally Important Change (MIC) has been defined in many ways [24], but we will use the concept “the smallest difference in score that patients perceive as important”.

In the present study, the patient global impression of change (PGIC) [25, 26] was used as the anchor for estimation of the MIC, this patient-reported outcome is the most appropriate to compare with another patient-reported outcome of HRQoL. The MIC was determined by the change observed in those patients who declared to be “minimally improved” at follow-up [24, 27–29].

#### 2. Distribution-based methods

This section refers to statistical techniques based on the distribution of scores, in order to provide information enough from different quantitative sources without reference of the patients’ point of view. The following parameters, whose formulas are shown in the Supporting information (S1 Text), were calculated:

*2*.*1*. *Magnitude of the change*. Although there are not standard or threshold values for these magnitudes, they furnish an intuitive approach to the importance of change (a higher change will be more important than a small one).

1. Intragroup difference follow-up (FU)-baseline. Negative figures reflect improvement and positive differences worsening, according to the PDQ-39 SI. This outcome is available in the primary study publication [14], but is shown here again for completeness.
2. Comparison of the magnitude of the difference FU-baseline inter-group [14].
3. Relative change or percentage of change (intragroup) [30] and inter-group comparison of proportions.

*2*.*2*. *Effect size*. Intra- and inter-group (paired and unpaired effect size, respectively) [31, 32].

*2*.*3*. *Threshold values and triangulation*. Comparison between the observed change and some thresholds proposed as representative of the MIC value (intra-group):

1. Standard error of the difference (S_*diff*_), as an estimate of the measurement error of change in longitudinal studies [33]. From this perspective, the S_*diff*_ (as the standard error of measurement, SEM) could be considered the threshold for a minimal important change [34, 35] in absence of the SEM, which could not be calculated due to the low number of stable patients at FU (n = 6).
2. Half a standard deviation at baseline [36, 37].

Different methods usually offer different results. Therefore, the calculation of an average value (“triangulation”) theoretically approaching the true MIC has been proposed [38, 39]. For this purpose, the values averaged were the anchor-based difference FU-baseline, the S_*diff*_, and ½ standard deviation at baseline.

#### 3. Distribution of benefit

3.1. Proportion of patients who improved ≥ 1 MIC, according to the average value.

3.2. Cumulative distribution function of responses [11].

3.3. Number needed to treat (NNT) for having one patient improved ≥1 MIC comparing DBS vs BMT groups [40, 41].

### Objective 2: Changes of disease characteristics associated with HRQoL changes

- Correlation (Spearman or Pearson coefficient) between change in the HRQoL scores and change in motor impairment, disability, mood, gait disorder, and social functioning were determined. Coefficient values ≥0.30 and ≥0.60 were considered as moderate and high correlation, respectively.
- Multiple regression analysis models were built to identify the reliability of the associations and their strength, with the change in HRQoL as dependent variable and the other changes (after exclusion of interaction and collinearity) as explanatory variables.

## Results

At baseline, the PDQ-39 SI of the 124 patients in the DBS arm was 30.18±14.1 (mean±SD), whereas it was 30.20±14.2 in the BMT group (n = 127). At 24-month follow-up, 120 patients remained in the DBS arm, with a PDQ-39 SI score of 22.40±1.41 (-7.8±1.2; p<0.001), and 123 patients in the BMT branch, with PDQ-39 SI of 30.44±1.40 (0.2 ± 1.1; p = 0.84) (inter-group difference, p = 0.002).

### Patient global impression of change

At FU, 120 with DBS and 122 BMT patients remained in the study and were evaluated with the PGIC. According to the PGIC, 107 (89.2%) patients in the DBS group reported improvement; 6 (5%) no change; and 7 (5.8%) worsening, whereas the corresponding values in the BMT group were 42 (34.4%), 10 (8.2%), and 70 (57.4%) (p<0.001). The distribution of the sample in the corresponding PGIC categories is shown in the Table 1. It is evident that for some categories, the small sample size conditions yield extremely wide (and not all that meaningful) confidence intervals.

**Table 1:** Patient global impression and related mean PDQ-39 SI change from baseline to 24 months.

	DBS		BMT	
Total n	120		122*	
PGIC level†	Number (%)	Mean [95% CI]	Number (%)	Mean [95% CI]
**Very much worse**	0 (0.0)	--	2 (1.6)	5.8 [-48.4, 60.1]
**Much worse**	3 (2.5)	14.8 [-11.4, 41.0]	26 (21.3)	9.9 [5.7, 14.1]
**Minimally worse**	4 (3.3)	-8.5 [-39.5, 22.5]	42 (34.4)	-0.7 [-3.8, 2.4]
**No change**	6 (5.0)	-7.5 [-23.5, 8.6]	10 (8.2)	0.5 [-7.0, 8.0]
**Minimally improved**	10 (8.3)	-5.8 [-9.9, -1.6]	15 (12.3)	-2.9 [-9.0, 3.1]
**Much improved**	59 (49.2)	-7.7 [-11.4, -4.1]	22 (18.0)	-4.8 [-9.4, -0.1]
**Very much improved**	38 (31.7)	-10.1 [-14.5, -5.8]	5 (4.1)	-14.1 [-26.4, -1.9]
**Much + Very much improved**	97 (80.8)	-8.7 [-11.4, -5.9]	27 (22.0)	-6.5 [-10.8, -2.2]

PGIC, Patient Global Impression of Change; CI, Confidence interval

* One BMT patient missing for the Patient Global Impression of Change.

† Categories of worse, no change, and improved are statistically significant between DBS vs. BMT (P<0.001).

Note: Negative mean changes indicate improvement as compared with baseline.

### Quantifying the meaning of health-related quality of life outcomes

Using the anchor-based method, the mean improvement in the group of patients who felt to be “minimally improved” with respect to the baseline was -5.8±5.8 (CI: [-9.9, -1.6]) in the DBS group vs -2.9±11.0 (CI: [-9.0, 3.1]) in the BMT group (p = 0.468).

Table 1 shows the mean PDQ-changes and confidence intervals as related to the PGIC-level. It is evident that the PDQ-39 SI change related to ‘much improved’ (-7.7 ± 14.1 for DBS and -4.8 ± 10.4 for BMT groups) and ‘very much improved’ (-10.1 ± 13.2 for DBS and -14.1 ± 9.9 for BMT groups) shows a gradation in which subjective level of improvement or worsening is associated with a stepwise change in HRQoL.

In the Table 2, the results of the distribution-based analysis in both treatment groups are shown. There was an evident change of PDQ-39 SI scores towards improvement in the DBS group, whereas change in the BMT group was towards impairment and negligible. The effect size value was ≥0.60 (“moderate”), both for the DBS group and inter-group difference, and negligible (0.02) for BMT. In regard to the Minimally Improved group in Table 2 the two threshold values (S_*diff*_ and ½ SD_baseline_) were, respectively, 6.0 and 7.1 for both the DBS group and the BMT group. Average of these values plus that obtained from the anchor in both groups produced a MIC estimate of 6.3 and 5.4 (in absolute values), respectively, indicating that the observed change (difference follow-up–baseline) was higher than the estimated MIC in the DBS group, but much lower in the BMT group.

**Table 2:** Data for interpretation of change in the PDQ-39 SI.

	DBS Group	BMT Group	Dif. Inter-group
Difference follow-up–baseline ^a^	-7.8 (1.2)	0.2 (1.1)	-8.0 (1.6)
*p*	<0.001	0.84	0.002
Relative change	-26%	1%	27%
Effect size [95%CI]	0.60	0.02	0.63 [0.37–0.89]
Minimally improved (MI)*			
N (% on the corresponding sample)	10 (8.3%)	15 (12.3%)	
Difference follow-up–baseline	-5.8	-3.0	
Standard error of the difference	6.0	6.0	-
½ standard deviation at baseline	7.1	7.1	-
Estimated MIC_MI_ ^b^	-6.3	-5.4	-
Proportion of patients improved ≥ 1 MIC_MI_	70/120 (58.3%)	39/122 (32.0%)	P<0.0001
NNT to improve ≥ 1 MIC_MI_	1.7	3.1	3.8
Much and Very much improved (M/VMI) *			
N (% on the corresponding sample)	97 (80.8%)	27 (22.0%)	-
Difference follow-up–baseline	-8.7	-6.5	-
Standard error of the difference	5.2	4.9	-
½ standard deviation at baseline	6.8	7.1	-
Estimated MIC_M/VMI_ ^c^	-6.9	-6.2	-
Proportion of patients improved 1≥MIC_M/VMI_	68/120 (56.7%)	37/122 (30.3%)	P<0.0001
NNT to improve ≥ 1 MIC_M/VMI_	1.8	3.3	3.8

^**a**^ Mean (standard error)

^b^ MIC_MI_: Minimally important change for minimally improved.

^c^ MIC_M/VMI_: Minimally important change for much and very much improved.

* According to the Patient Global Impression of Change.

**Fig 1:**
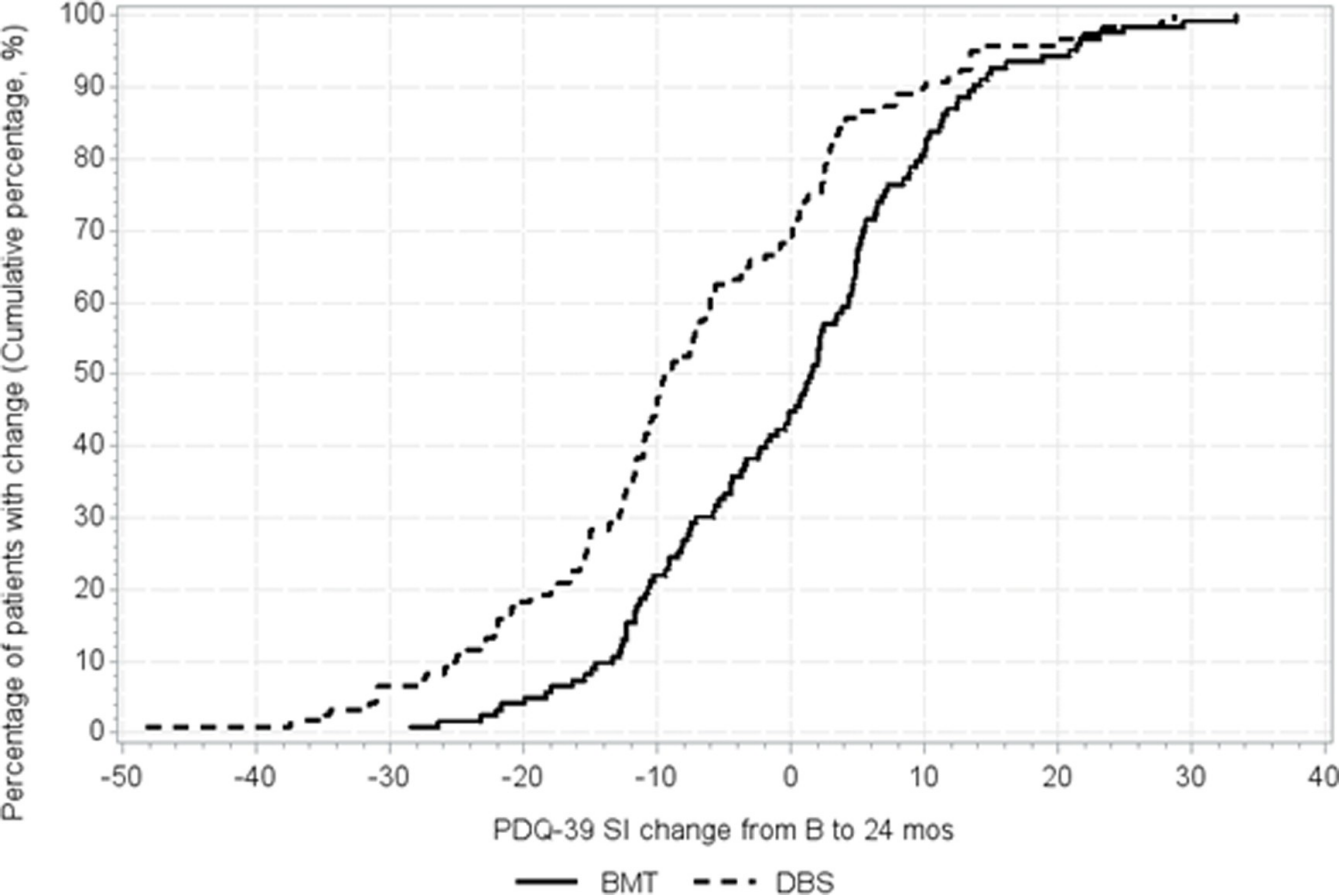
Cumulative distribution curves of PDQ-39 change from baseline to 24 months (1-cumulative %).

The proportion of patients who minimally improved at least 1MIC in the DBS group was 58.3% (70/120), whereas only 32.0% (39/122) improved in the BMT group (p<0.0001) (Table 2). Subsequently, the NNT to observe one patient improving the MIC or more was 1.7 for DBS and 3.1 for BMT. As most (80.8%) of the DBS patients were ‘much or very much improved’, similar calculations were carried out for these subjects, collapsed to a unique level (Table 2). The MIC for this level ‘much or very much improved’ was 6.9 for DBS and it was reached or surpassed by 56.7% (68/120) of subjects in this group, whereas it was 6.2 for BMT, achieved by 30.3% (37/122) of patients in this group (p<0.0001). The corresponding NNT were similar for ‘minimally improved or more’ and ‘much or very much improved” (1.7 vs 1.8 for the DBS group; 3.1 vs 3.3 for BMT). The NNT for DBS compared to BMT was 3.8 (Table 2). Fig 1 shows the cumulative distribution function for both groups in the study, with more patients showing benefit of the respective MIC value in the DBS group.

### Changes of disease characteristics associated health related quality of life changes

The change in the PDQ-39 SI was highly correlated with the improvement in psychosocial adjustment (SCOPA-PS) and depression (BDI), and a moderate association was found with the change in the activities of daily living (ADL, UPDRS II) and pain (VAS-Pain). The correlation was moderate/weak with SOFAS. Most of the classical motor parameters like UPDRS III in the worst or best condition, OFF-time or fluctuations and dyskinesia, were only weakly correlated (Table 3).

**Table 3:** Correlations between change in PDQ-39 and change in other scales for the DBS group *.

Endpoint change from baseline to 24 months	Spearman R	[95% CI]	P-value
SCOPA-Psychosocial	0.69	[0.58, 0.77]	<0.0001
Beck Depression Inventory	0.67	[0.56, 0.76]	<0.0001
MOS Short Form 36 items (SF-36)	0.54	[0.40, 0.66]	<0.0001
UPDRS II "worst"	0.42	[0.26, 0.56]	<0.0001
Visual Analog Scale–Worst pain	0.31	[0.14, 0.46]	0.0006
SOFAS	0.30	[0.12, 0.45]	0.0011
Starkstein’s Apathy Scale	0.26	[0.08, 0.42]	0.0043
PIGD (On stim/On med)	0.24	[0.06, 0.41]	0.0088
“On time” without troublesome dyskinesias	0.23	[0.04, 0.41]	0.0208
UPDRS III (On stim/Off med)	0.22	[0.04, 0.39]	0.0198
UPDRS III (On stim/On med)	0.18	[-0.01, 0.35]	0.061
“Off time” from motor diary	0.16	[-0.04, 0.35]	0.109
UPDRS IV	0.08	[-0.1, 0.26]	0.3715

SCOPA, Scales for Outcomes in Parkinson’s Disease; UPDRS, Unified Parkinson’s Disease Rating Scale; SOFAS, Social- and Occupational Functioning Assessment Scale; PIGD, Patient Global Impression of Change

*Coefficient values ≥0.30 and ≥0.60 were considered as moderate and high correlation, respectively.

Table 4 shows the results of the regression model built to identify determinants of change in QoL (PDQ-39 SI), which was the dependent variable. To this purpose, the independent variables in the model were selected after discarding collinearity (intercorrelation coefficients <0.75). Change in SCOPA-PS and SF-36 were not included due to their direct interaction with the dependent variable, and "On time without troublesome dyskinesias" was discarded by potential interaction with UPDRS-IV.

**Table 4:** Model estimates from linear regression for the change in PDQ-39 SI.

Prob>F: P<0.0001	Parameter Estimate	Standard Error	t Value	Pr > |t|	Standardized Estimate
Adjusted R^2^: 0.4679					
*Intercept*	*2*.*43*	*1*.*62*	*1*.*5*	*0*.*136*	*0*
**Beck Depression Inventory**	0.791	0.15	5.12	<0.0001	0.411
**UPDRS III (On stim/On med)**	0.004	0.16	0.02	0.982	0.002
**UPDRS II "worst"**	0.647	0.18	3.63	0.0004	0.302
**Visual Analog Scale—Worst pain**	0.845	0.29	2.89	0.0047	0.226
**UPDRS IV**	0.064	0.32	0.2	0.844	0.015
**Starkstein’s Apathy Scale**	0.304	0.21	1.46	0.148	0.115
**SOFAS**	-0.061	0.08	-0.76	0.448	-0.060

The independent variables represent the change observed in each of the listed scales UPDRS, Unified Parkinson’s Disease Rating Scale; SOFAS, Social- and Occupational Functioning Assessment Scale.

## Discussion

The EARLYSTIM Study showed a favorable outcome at FU in the primary endpoint, HRQoL measured with the PDQ-39 (improvement of 26%), in the group treated with DBS whereas the BMT group slightly worsened (1%) [14]. In this pivotal study, information about the effect of the intervention on HRQoL domains, UPDRS parts II to IV, psychosocial adjustment, neuropsychiatric disorders, and levodopa-equivalent daily dose also showed a significant beneficial effect on these endpoints for the DBS group. However, neither the importance that the change in HRQoL entailed for the patient nor the relationship between this outcome and the change in the other variables in the study were explored. The present analysis was aimed, therefore, to investigate these gaps.

### Objective 1

Two approaches are used for interpretation of change in patient-reported outcomes, in general, and HRQoL in particular: anchor-based and distribution-based methods [11, 28, 29]. The only finding of an anchor-based method we obtained was the difference baseline-FU for the group of patients who declared to be “minimally improved” (5.8 points for the DBS and 2.9 points for the BMT group). However, the number of patients in this situation was quite small (10 DBS and 15 BMT patients), representing 8.3% and 12.3% of the respective total samples (Table 1). These figures are insufficient for providing reliable information about the MIC, although they would indicate a significant benefit in the DBS group only (Table 2). Given that most of DBS patients declared to be ‘much/very much improved’, the same procedure that for ‘minimally improved’ category was performed for those patients, and the mean difference FU-baseline was -8.7 [-11.4, -5.9] in the DBS versus -6.5 [-10.8, -2.2] in the BMT group.

A variety of parameters with the distribution of score changes was subsequently calculated. Anchor-based methods are preferred [11, 42] because they connect the concept measured by the patient-reported outcome with the anchor, making easy and reliable the interpretation of the outcome. Distribution-based methods, on the contrary, have not a connection with a directly interpretable measure and mainly provide information based on the magnitude of the change. This, however, exceeds the information of a merely ‘statistically significant difference’. Both approaches, anchor- and distribution-based methods, have advantages and disadvantages [28, 39, 42–44] (S1 Table).

Summarizing the results at FU of the distribution-based methods (Table 2):

1. The relative change and effect size showed a moderate benefit of DBS on HRQoL, whereas a slight impairment was observed in the BMT group.
2. The observed change (-7.8) was higher than the MIC estimated value for a minimal (-6.3) or even a better improvement (-6.9) in the DBS group, but not in the case of the BMT (0.2 vs. -5.4 and -6.2), respectively.
3. Almost 90% of the patients in the DBS group declared improvement (58.3% and 56.7% of them beyond the estimated MIC for this arm), whereas in the BMT group these proportions were 32.0% and 30.3%, respectively.
4. In both treatment groups there were patients declaring ‘much’ or ‘very much” improvement, but the proportions were significantly different (81% for DBS, 22% for BMT), as well as the proportion of patients who improved more than their respective minimal change for much and very much improved (56.7% for DBS, 30.3% for BMT).
5. The comparative NNT to improve ≥1 minimal change for much and very much improved by DBS vs BMT was 3.8.

According to these results at long-term, STN-DBS generated benefit in HRQoL in the vast majority of patients and such improvement was considerable in almost 60% of them (Fig 1). On the contrary, almost two thirds of patients on BMT were stable or worse at FU in the BMT group of patients.

Using a transition question as anchor, Peto et al. [45] carried out a postal survey with 728 responses (53.1% of the baseline sample) from members of the UK Parkinson’s Disease Society, at 6-month of the first evaluation with the PDQ-39. The mean change (impairment) in patients who declared to be “a little worse” (n = 192) was 1.60 (±8.89), with an effect size 0.10.

Fitzpatrick et al [33] determined by distribution-based methods (SEM and S_*diff*_) the threshold for a real change and, next, compared these results to those from the anchor-based approach to explore the relationship between both methods. They found that one SEM values were relatively close to the MIC values obtained with the anchor-based, in turn usually smaller than the S_*diff*_. If SEM represents the minimal change beyond the measurement error, it constitutes the lower threshold for considering a change as real and the S_*diff*_, which is higher than the SEM, could be nearer of the MIC than the SEM (the limit of the “noise” by error). In the clinical sample of this study, eight patients declared improvement at follow-up (4 months), with a change in the PDQ-39 SI of– 2.15 (±6.62) points, whereas in 40 patients considered to be worse the change was 3.31 (±8.80). The S_*diff*_ was 5.39, clearly higher than the change observed, and quite close to the S_*diff*_ values found in the present analysis (6.0 for both arms). In the present study, the SEM based on agreement (intraclass correlation coefficient of stable patients) [46] was not used due to the very small size of this group in both arms.

In another series of PD patients followed-up for one year by Martinez-Martin et al. [47], the sample tended to worsen and the SEM for the PDQ-39 SI was 4.26 (baseline) and 4.77 (follow-up). Horváth et al. [48] found, through a transition question, a minimal clinically important difference (MCID) for improvement of– 4.72 points (–5.28 for patients with moderate; –4.17 for patients with severe PD), effect size of 0.25, in a cohort of 365 PD patients in “regular care”, with a median of 4 follow-up visits at median intervals of 6-month intervals. The corresponding values for worsening in this study were: 4.22 points (moderate = 4.40; severe = 4.99), and effect size of 0.23. These MCID figures are very close to the SEM estimated by Martinez-Martin et al. [47] and agree with observations and proposals about the similarity between MID and SEM [34–36, 43].

Holden et al. [49] also used a transition question, the Clinical Global impression of Change, and determine the MCID (improvement) in 90 patients with parkinsonism, after a palliative intervention clinical trial with six months follow-up. The absolute MCID obtained for the PDQ-39 (12.7), was considered valid by the authors for this kind of populations. Similar to our findings, a decrease in the PDQ-39 SI score was unexpectedly observed in patients with minimal worsening, a finding probably related with “the full spectrum of patient experience” [49] and expectations [50].

From the previous considerations, it is concluded that MIC varies with the populations, disease severity, type of study (natural progression of disease vs. intervention), and length of the follow-up. Therefore, comparison with other studies performed in similar circumstances is not possible if these studies do not exist, like in the case with the EARLYSTIM study.

Concerning the effect size, most studies with conventional levodopa or dopamine agonists showed changes from 5–30% and weak to moderate effect sizes, whereas therapies for advanced patients achieved 30–50% and moderate to large effect sizes [51–53]. Several reviews about the effect of the bilateral STN-DBS on the HRQoL, measured with the PDQ-39, found improvements of 19–34%, with effect sizes 0.60–0.80 [51, 52, 54]. Therefore, the results of the intervention in the present study, are in line with the published literature.

### Objective 2

This analysis was performed to identify how the change in other variables captured in the study could be associated with the change in HRQoL. A close correlation was found with improvement in psychosocial adjustment and depression; moderate with improvement in the global HRQoL, activities in daily living, and pain; and a weak association was observed with the other variables (Table 3). According to the results of the multiple regression, the improvement in depression, functional state, and pain were independent and significant determinants of the change in HRQoL.

There are many factors associated with and able to influence the HRQoL of PD patients [2–4, 55–59]. Depression, disability, and pain are universal determinants of HRQoL, and they are very frequently present and combined in PD patients, causing a severe deterioration of their HRQoL. If these factors remain significantly improved at long-term after surgery, it would be expected that their improvement entailed a significant improvement in patients’ quality of life. Typically, patients have received DBS therapy due to motor complications. The current analysis as well as the data from the main study may prompt considerations about non-motor and quality-of-life aspects to become indication criteria for DBS. More studies are needed here.

## Conclusions

From the previous discussion it may be concluded that DBS improved patients’ HRQoL in a significant and moderate degree, as a whole, over a two year follow up period. In this group, the beneficial effect was present in the vast majority of patients. These results are even more remarkable when compared to the BMT group, which tended to worsen their HRQoL over the observation time. MIC of the PDQ-39 SI for both populations in the study was determined by means of anchor- and distribution-based methods.

## Supporting information

S1 Text Applied distribution-based methods. Formulas.
(DOCX)

S1 Table Advantages and disadvantages of the methods for interpretation of outcomes. (DOCX)
