# Supplementary material for: Interpretation of health-related quality of life outcomes in Parkinson’s disease from the EARLYSTIM Study
Source: PLoS One. 2020 Aug 21;15(8):e0237498. doi: 10.1371/journal.pone.0237498 (PMC7442251; doi:10.1371/journal.pone.0237498)
Supplement: S1 Text — Formulas. (DOCX) [file pone.0237498.s001.docx]

**PONE-D-20-09678 Supportive information**

**S1**

**Distribution-based methods**

***Formulas applied***

2.1. Magnitude of the change.

2.1.1. Intragroup difference follow-up (FU)-baseline: Mean_FOLLOW-UP_ – Mean_BASELINE_

2.1.2. Comparison of the magnitude of the difference FU-baseline inter-group: Mixed model statistical analyses with normality assumption with the baseline value for baseline adjustment, main effects for group and time, a group-by-time interaction term, center as random effect, and a generalized covariance matrix to account for serial dependency among observations.

2.1.3. Relative change or percentage of change (intragroup):

Mean_FOLLOW-UP_ – Mean_BASELINE_

Mean_BASELINE_

(Deyo and Centor. J Chronic Dis. 1986; 39: 897-906) [29]

2.2. Effect size

- A. Effect size intra-group (paired effect size):

*d*z = Mz / SDz

Where *d*_Z_ is the effect size

M_Z_ is the mean of the difference follow-up–baseline

SD_Z_ is the standard deviation of the difference follow-up–baseline

- B. Effect size for the difference between groups:

M_Z_ DBS – M_Z_ BMT

SD_Z_ _pooled_

Where M_Z_ is the mean of the difference follow-up–baseline

SD_Z pooled_ = √ (SD_Z DBS_^2^ + SD_Z BMT_^2^) / 2

(Nakagawa and Cuthill. Biol Rev Camb Philos Soc. 2007; 82: 591-605) [30]

(D. Lakens. Front Psychol. 2013; 4: 863) [31]

2.3. Threshold values and triangulation.

2.3.1. Standard error of the difference:

S*_diff_* = √ (SEM_1_^2^ + SEM_2_^2^)

Where SEM_1_ and _2_ represent SEM at baseline and follow-up

SEM_1_ = SD_1_ * √1 – alpha_1_

SEM_2_ = SD_2_ * √1 – alpha_2_

SD_1_ and _2_ represent the SD at baseline and follow-up

Alpha_1_ and _2_ is the reliability coefficient at baseline and follow-up

(Fitzpatrick et al. J Clin Epidemiol. 2004; 57: 40-44) [32]

3.3. NNT: 1 / %P_MIC_

Where %P_MIC_ is the proportion of patients improved ≥ 1 MIC

NNT comparing DBS vs. BMT:

1

NNT =

(%P_MIC-DBS_) – (%P_MIC-BMT_)

(Norman et al. Med Care. 2001; 39:1039-47) [39]

(Wyrwich et al. Qual Life Res 2005;14:285-295) [40]
