# Supplementary material for: Interpretation of health-related quality of life outcomes in Parkinson’s disease from the EARLYSTIM Study
Source: PLoS One. 2020 Aug 21;15(8):e0237498. doi: 10.1371/journal.pone.0237498 (PMC7442251; doi:10.1371/journal.pone.0237498)
Supplement: S1 Table — (DOCX) [file pone.0237498.s002.docx]

**S1 Table. Advantages and disadvantages of the methods for interpretation of outcomes**

|  | **Anchor-based methods** | **Distribution-based methods** |
| --- | --- | --- |
| **Framework** | Require an independent and meaningful standard (anchor), clinically relevant | Relate the changes in PRO scores to variability parameters and describe the observed differences according to standard values |
| **Advantages** | The change in the PRO is directly interpretable due to the association with the anchor | Determine if change is beyond random variation  Provide results with equivalent meaning across measures, populations, and studies |
| **Disadvantages** | The anchor may be inappropriate  Recall biases by retrospection  Arbitrary assignation of the level of change (minimal, moderate, large)  Use of global ratings, with potential loss of information (variance)  The sensitivity of the measure is not taken into account  Difficulties when the relationship anchor-PRO is not lineal | Do not provide direct information about importance of the change  There are few standard criteria for clinical significance  Some methods determine the minimal detectable change rather than the minimal important change |
| **Recommendation** | Preferred | Support |
